# Immunophenotype of T Cells Expressing Programmed Death-1 and Cytotoxic T Cell Antigen-4 in Early Lung Cancer: Local vs. Systemic Immune Response

**DOI:** 10.3390/cancers11040567

**Published:** 2019-04-21

**Authors:** Iwona Kwiecien, Tomasz Skirecki, Małgorzata Polubiec-Kownacka, Agata Raniszewska, Joanna Domagala-Kulawik

**Affiliations:** 1 Department of Internal Medicine and Hematology Laboratory of Flow Cytometry, Military Medical Institute, Warsaw, ul. Szaserow 128, 04-141 Warsaw, Poland; kwiecieniwi@gmail.com; 2 Laboratory of Flow Cytometry, Centre of Postgraduate Medical Education, ul. Marymoncka 99/103, 01-813 Warsaw, Poland; 3 Department of Surgery, Institute of Tuberculosis and Lung Diseases, ul. Plocka 26, 01-138 Warsaw, Poland; m.polubiec@igichp.edu.pl; 4 Department of Pathology, Medical University of Warsaw, ul. Pawinskiego 7, 02-106 Warsaw, Poland; agataraniszewska@vp.pl; 5 Department of Internal Medicine, Pulmonary Diseases and Allergy, Medical University of Warsaw, ul. Banacha 1a, 02-097 Warsaw, Poland; domagalakulawik@gmail.com

**Keywords:** CTLA-4, cytotoxic T cell antigen 4, PD-1, programmed death-1, BALF, bronchoalveolar lavage fluid, lung cancer, immunotherapy

## Abstract

The overexpression of programmed death-1 (PD-1) and cytotoxic T cell antigen 4 (CTLA-4) receptors on T cells are among the major mechanisms of tumor immunoevasion. However, the expression pattern of these receptors on T cell subpopulations of a different activation status and at different sites is poorly characterized. Thus, we analyzed the expression of PD-1 and CTLA-4 on the naïve, activated, memory, and activated memory T cells. Bronchoalveolar lavage fluid (BALF) from the lung affected by lung cancer (clBALF), the opposite ‘healthy’ lung (hlBALF), and peripheral blood (PB) samples were collected from 32 patients. The cells were analyzed by multiparameter flow cytometry. The proportion of memory, activated, and activated memory CD8+ cells with the expression of PD-1 and CTLA-4 were elevated in the clBALF when compared to the hlBALF (insignificantly), but these proportions were significantly higher in the BALF when compared with the PB. The proportions of PD-1+ and CTLA-4+ T cells were elevated in the squamous cell carcinoma when compared to the adenocarcinoma patients. Also, the expression of PD-1 and CTLA-4 on T cells from the BALF was significantly higher than from PB. We report for the first time the differential expression of checkpoint molecules on CD4+ and CD8+ lymphocytes at a different stage of activation in the local environment of lung cancer. Moreover, the circulating T cells have a distinct expression of these receptors, which suggests their poor utility as biomarkers for immunotherapy.

## 1. Introduction

Lung cancer is a serious oncological problem worldwide. It is the leading cause of death among cancer patients. However, the efficacy of the new immunotherapy methods with check-point inhibitors (ICIs) has been recently demonstrated in about 40% of patients with non-small cell lung cancer (NSCLC) [1,2,3,4]. T cells play a key role in anticancer defense, but their population is modulated in the course of cancer [5,6]. The numerous suppressory and regulatory mechanisms inhibit the recognition of lung cancer antigens and are capable of blocking the lymphocyte activation. The goal of lung cancer immunotherapy is to improve the cytotoxic effect of lymphocytes by inhibiting suppressory molecules, such as: programmed death-1 (PD-1) and cytotoxic T cell antigen 4 (CTLA-4). 

PD-1 has an essential role in balancing protective immunity and immunopathology, homeostasis and tolerance. T cell activation is a highly regulated process involving the peptide—MHC engagement of the T cell receptor and positive costimulatory signals. Upon activation, co-inhibitory ‘checkpoints’, including PD-1, become induced to regulate T cells. However, during responses to chronic infections and tumors, PD-1 expression can limit protective immunity [7]. PD-1 can be expressed by a variety of immune cells including T lymphocytes: CD4+ T cells, CD8+ T cells, B lymphocytes, natural killer (NK) cells, activated monocytes, dendritic cells (DCs), and macrophages [7,8]. Because of the persistent exposure to antigens, PD-1 is selectively upregulated in T cells; thus, the expression of PD-1 is one of the makers of exhausted T cells [9,10]. However, little is known about the differential expression of PD-1 on other types of T cells like memory, naïve, and activated ones in the tumor microenvironment. The PD-1/PD-L1 pathway plays a critical role in cancer immunology. Blocking antibodies against these molecules provide benefits in clinical trials and in practice. The introduction of antibodies blocking the PD-1 receptor (or with the anti-CTLA4 checkpoint inhibitor) has improved survival profiles and acquired high response rates in several solid tumors [11,12].

CTLA-4 plays a crucial role in the suppression of the immune anticancer response. CTLA-4, also known as CD152, is a protein receptor that downregulates the T cell response. CTLA-4 binds to CD80 or CD86 on antigen-presenting cells transmitting an inhibitory signal to T cell [13]. The cell membrane CTLA-4 undergoes continuous recirculation via clarithin-mediated endocytosis and the majority of CTLA-4 is localized in the endosomes. Thus, there are two forms of CTLA-4 expression: on the cell surface and the intracellular one [13]. CTLA-4 is constitutively expressed on T regulatory cells (Tregs), but not on other resting naïve T cells [14]. However, both CD4 and CD8 T cells can express it on the cell surface upon activation [15]. Interestingly, especially activated Th17 cells express high level of surface CTLA-4 comparing to the naïve and memory Th1 cells [16]. mRNA for CTLA-4 in conventional T cells is induced very rapidly (within 1 hour [17]) after the TCR engagement followed by the surface CTLA-4 expression which peaks in fully activated T cells approx. 48 hours later [18,19,20]. Also, IL-2 and IFN-γ have the ability to induce CTLA-4 expression [20]. We previously demonstrated a significantly elevated proportion of CTLA-4+ Tregs in the lung cancer environment assessed by BALF analysis, when compared with the systemic compartment [21].

The studies on the role of CTLA-4 and PD-1 expression on T cells in cancer were mainly based on the analyses of circulating T cells [22,23,24]. Some data come from the investigations of tumor infiltrating lymphocytes (TIL) in resected tumors [25]. Previously, we presented the results of the studies on bronchoalveolar lavage (BAL) cells showing the usefulness of this method in the examination of local immune response in the lung cancer TME [21,26,27,28]. BAL may be performed in lung cancer patients even in the advanced stages of the disease as part of the diagnostic procedure. BAL enables the retirement of the full spectrum of immune cells from alveoli and bronchioles that can be further analyzed. The aforementioned reasons prompted us to choose the BAL fluid (BALF) as a basic material for this research. 

The aim of this study was to evaluate PD-1 and CTLA-4 expression on T cells in a different maturation status: from naïve cells to memory activated T cells in the lung cancer TME and systemic circulation. It was based on the examination of BALF from the lung affected by cancer (clBALF as the local environment), compared to the opposite ‘healthy’ lung (hlBALF as the internal control) and the peripheral blood (PB reflecting the systemic changes) from the same patient. We investigated the proportions of T cell subpopulations expressing PD-1 and CTLA-4 in these three compartments and then we evaluated the relations between them. The phenotypic characterization of these crucial regulatory molecules on the different types of T cells in the close lung cancer microenvironment evaluated by BALF examination may improve the understanding of the conditions of immunotherapy action. 

## 2. Results

### 2.1. Patients

Finally, the studied group consisted of 21 patients with primary NSCLC and 11 patients with other lesions (benign tumors, inflammatory lesions, or metastases from other primary tumors). The stage of the lung cancer was established according to the seventh TNM classification, the histological type according to the WHO 2015 classification. Clinical data are shown in Table 1. The group with benign lesions (inflammatory tumors: 7, hamartoma: 1, tuberculoma: 2) consisted of three women and eight men, in the mean age 64.5 ± 9.4 years. Three patients were ever smokers with mean pack-years: 27.3 ± 12.2. For further analysis, two patients with metastases from other than lung cancer primary site were excluded from this group. 

### 2.2. BALF

A routine BAL cells analysis did not reveal any significant differences between the cancerous and opposite ‘healthy’ lung. The total cell count was (2.7 ± 2.5) × 10^6^ in the clBALF and (2.9 ± 2.6) × 10^6^ in the hlBALF, respectively. The inflammatory cells proportion in all BALF samples was in normal ranges [29]. We did not find any malignant cells in the BALF slides stained with hematoxylin- eosin. 

The example of BALF flow cytometric analysis is presented in Figure S1. The proportion of CD4+ cells and the CD4+/CD8+ ratio differed significantly between the BALF and PB (Table 2). The proportion of naïve CD8+ and CD4+ cells was significantly higher in the PB than in the BALF (6.5-fold and 2.7-fold, respectively), unlike the memory and activated CD8+ and CD4+ cells which were in a significantly higher proportion in the BALF than in PB. 

### 2.3. PD-1 Expression Pattern

**Figure 1:**
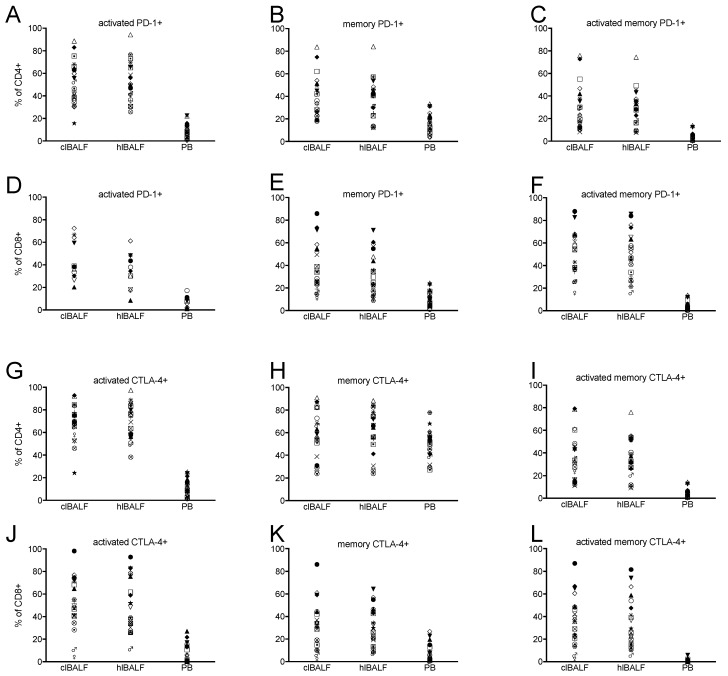
PD-1 and CTLA-4 expression on T cells from lung cancer patients. Data presented as individual plots of results from each patient obtained from lung cancer BAL (clBALF), the opposite ‘healthy’ lung BAL (hlBALF) and the peripheral blood (PB) Proportions of: (**A**) activated PD-1+ CD4+ T cells; (**B**) memory PD-1+ CD4+ T cells; (**C**) activated memory PD-1+ T cells; (**D**) activated PD-1 CD8+ T cells; (**E**) memory PD-1+ CD8+ T cells; (**F**) activated memory PD-1+ T cells; (**G**) activated CTLA-4+ CD4+ T cells; (**H**) memory CTLA-4+ CD4+ T cells; (**I**) activated memory CTLA-4+ T CD4+ T cells; (**J**) activated CTLA-4+ CD8+ cells; (**K**) memory CTLA-4+ CD8+ cells; (**L**) activated memory CTLA-4+ CD8+ T cells.

**Figure 2:**
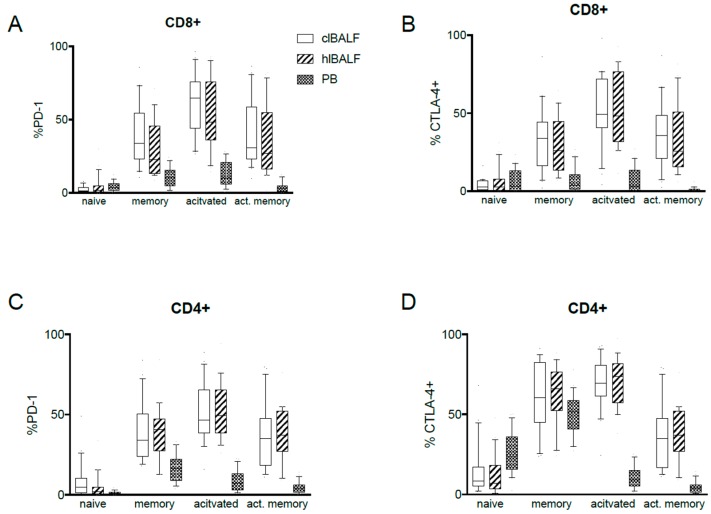
PD-1 and CTLA-4 expression by T cell subsets in different compartments. Differences between the proportions of naïve (n), memory (m), activated (a), and activated memory (am): (**A**) CD8+ PD-1-positive cells; (**B**) CD8+ CTLA-4-positive cells; (**C**) CD4+PD-1-positive cells; and (**D**) CD4+CTLA-4-positive cells in the clBALF, hlBALF and PB.

We found the expression of PD-1 on each investigated subset of CD8+ as well as CD4+ cells in both BALF and PB. There was a significantly higher proportion of all CD8+ and all CD4+ cells with PD-1 expression in the BALF when compared with the PB (Figure 1A–F and Table 3). Also, the frequency of PD-1 intermediate CD4+ T cells was higher in the BAL from cancer affected lung than peripheral blood while frequency of PD-1 low cells was lower in clBAL (Supplementary Materials Figure S2). BALF CD8+ T cells contained more PD-1 high and intermediate cells than PB. This was related with lower percentage of PD-1 low cells (Supplementary Materials Figure S2). The proportion of memory, activated and activated-memory CD8+ and CD4+ cells with expression of PD-1 in the BALF was higher than in the PB (Table 3 and Figure 2A,C). The proportion of naïve CD8+ cells with PD-1 expression was higher in the PB. In the comparative analysis between the clBALF and hlBALF, we observed a higher proportion of memory CD8+PD-1+, activated CD8+PD-1+, activated-memory CD8+PD-1+ cells in the clBALF when compared with the hlBALF (differences not significant). On the contrary, for CD4+ cells the proportions of memory and activated CD4+PD-1+ cells were slightly lower in the clBALF, but the prevalence of naïve CD4+PD-1+ cells was higher in the clBALF than in the hlBALF, *p* = 0.07. Next, we evaluated the geometric mean fluorescence (GMF) intensity of PD-1 and we found significant differences between the BALF cells and the PB (Table 3). The GMF intensity of PD-1+ on naïve CD8+ cells and memory CD4+ cells was lower in the PB than in the BALF. For activated and activated-memory CD8+ and CD4+ cells’ GMF, the intensity of PD-1 was significantly higher in the PB than in the BALF. No differences in the GMF intensity of PD-1 on CD8+ and CD4+ cells between the clBALF and hlBALF were found. 

### 2.4. CTLA-4 Expression Pattern

We observed the expression of CTLA-4 on each analyzed subpopulation of CD8+ as well as CD4+ cells. About half of CD8+ BALF cells expressed CTLA-4 while only one sixth of PB cells were CTLA-4 positive (Table 4). We did not find any differences in the frequencies of CTLA-4 hi/intermediate/low T cells between the analyzed compartments. There was a significantly higher proportion of memory, activated, and activated-memory CD8+ cells with the expression of CTLA-4 in the BALF when compared with the PB (Table 4, Figure 1B). In the analysis of differences between the clBALF and hlBALF we observed only a higher proportion of activated-memory CD8+CTLA-4+ cells in the clBALF when compared with the hlBALF (differences not significant, Figure 2B). CD4+ cells were found to bear CTLA-4 in the BALF and PB. The proportion of naïve CD4+/CTLA-4+ cells was significantly higher in the BP than in the BALF, while the proportion of memory and activated memory CD4+CTLA-4+ cells was significantly elevated in the BALF (Figure 2D). 

The geometric mean fluorescence (GMF) intensity of CTLA-4 was significantly higher on CD8+ and CD4+ cells and on their subpopulations in the PB when compared with the BALF (Table 4). No differences in the GMF intensity of CTLA-4 on CD8+ and CD4+ cells between the clBALF and hlBALF were found. 

### 2.5. PD-1 and CTLA-4 Expression in Relation to Clinical Data

The proportion of CD8+PD1+ activated cells correlated with almost all subpopulations of CD8+ and CD4+ cells with PD-1 and CTLA-4 expression (Supplementary materials Table S1).

**Figure 3:**
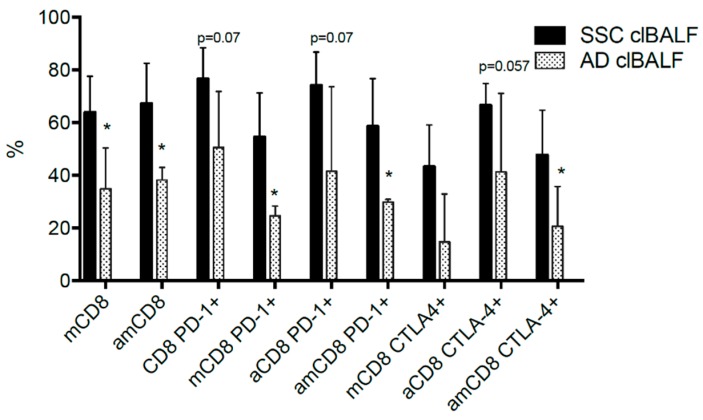
Significant differences in the proportion of clBALF cells with the expression of PD-1 and CTLA-4 between squamous cell carcinoma (SSC) and adenocarcinoma (AD) (* *p* < 0.05, exceptions were shown). Abbreviations: n—naïve, m—memory, a—activated, and am—activated memory cells.

We found significant differences in the proportion of analyzed cells when the two main histological types of NSCLC were compared: squamous cell carcinoma (SSC) and adenocarcinoma (AD). Generally, the proportions of PD-1+ and CTLA-4+ T cells and PD-1+ and CTLA-4+ GMF were higher in the clBALF from the patients with SSC than in the ones with AD. As for the whole study group, we found no significant differences between the tumor-affected lung and the opposite lung when we analyzed the SSC and AD subgroups. In Figure 3, we present all statistically significant differences which were observed in the population of CD8+ cells; for CD4+ cells no significance was found. 

The influence of tobacco smoke on the expression of PD-1 and CTLA-4 was difficult to assess as almost all patients were ever smokers. Based on the analysis of correlation between the cell profile and the smoking history we mostly found a significant reversed correlation between the proportion of PD-1+ and CTLA-4+ cells with the number of pack years smoked in the hlBALF and PB (Supplementary materials Table S2). 

We did not find any relation of BALF cell profile with EGFR mutation. 

Finally, we compared the cell profile in the BALF and PB of patients with lung cancer and benign lesions. As presented in the Table S3, the proportion of cells with PD-1 and with CTLA-4 expression was the highest in the BALF harvested from the cancer site. Supplementary materials Figure S2 and Table S3 present the proportion of PD-1+ and CTLA-4+ cells in the BALF and PB of patients with benign lung lesions (Supplementary materials Table S4 and Figure S3). The GMF of cells from the patients with benign lesions did not differ when compared with the lung cancer patients. 

## 3. Discussion

Immunotherapy with immune check-point inhibitors (ICIs) has brought real progress in the treatment of solid tumors including lung cancer. A proper qualification to this therapy presents a real challenge. To date, the only approved biomarker for anti-PD-1 agents is the degree of the expression of PD-L1 on tumor cells, but the novel biomarkers beyond PD-L1 are widely investigated. In our study, we show for the first time the differential expression of check-point molecules: PD-1 and CTLA-4 on CD8+ and CD4+ cells at the various stages of maturation and differentiation in the lung cancer microenviroment, e.g., bronchoalveolar lavage fluid (BALF) harvested form the tumor proximity. In contrast to the majority of studies on T cells in lung cancer, we took advantage of the BAL technique instead of analyzing the tumor samples. We argume that BAL is a non-invasive procedure that can be safely performed at any stage of the diagnostic/therapeutic process. What we also find important is that BALF reflects the cellular composition of the alveolar compartment not only within the tumor itself, but also its microenvironmental niche. The most striking findings of this study are: the difference of the proportion of PD-1 and CTLA-4 positive cells in the BALF when compared with the peripheral blood in the same patient and the elevated proportions of activated CD8+ cells with PD-1 and CTLA-4 molecules in the BALF from the cancer environment, especially in squamous cell carcinoma. For the first time, we used a direct comparison of the immune cells from the BALF affected by cancer with the opposite ‘healthy’ BALF. 

We used the commonly-applied markers for the analysis of naïve, activated, and memory T cells. CD69 is a marker of early activation and CD127 is a marker of antigen-experienced memory T cells. The presence of naïve cells in the blood and the predominance of activated memory cells in the lung observed in this study is not surprising. Tissue T cells with a phenotype of end-of-the-life and the resident memory (rm) lung cells are described [30]. These rm T cells are the precursors of exhausted (ex) T cells [31] and are characterized by the expression of CD69. The latter are unable to mediate cytotoxicity, but the re-invigoration of ex T cells is possible [9,32]. In this study, the activated memory T cells correspond to the rm T cells. The potential of reinvigoration is a newly recognized function of ICIs and may serve as a predictive factor in this therapy [32]. Of the two main T cell subtypes CD8+ cells are engaged to a greater extend in the anticancer response. The pre-existing CD8+ rich infiltrates in solid tumors are the indicators of a good prognosis and response to ICIs [33,34,35]. In the recent study, Djenidi et al. described the phenotype of TIL in lung cancer being in the majority activated, memory CD8+ cells [36]. We previously showed a significant augmentation of CD8+ cells in the BALF of cancer patients than of healthy subjects [37]. Also in this study, the changes of check-point molecules expression concern mainly the CD8+ population. 

In this study, we investigated three compartments of the immune response: a lung with cancer (clBALF), the opposite lung (hlBALF), and peripheral blood. Interestingly, we did not find significant differences in the analyzed T cell subpopulations between the clBALF and hlBALF. It may indicate that both lungs form an integrated functional system. Similar results were presented by Zicos et al., who used a procedure similar to ours [38]. In their study, the proportion of naïve, memory, and effector CD4+ and CD8+ cells was similar in the BALF from lung affected by cancer when compared with the contralateral lung. Also, in our unpublished study, we found no differences in the panel of cytokines concentration in the BALF from cancerous lung vs. the opposite one apart from some cytokines with regulatory function (ERS Congress, 2017). On the contrary, in our other studies we found significant differences between both lungs: the proportions of T regulatory cells (Tregs), CTLA-4+ Tregs, and M2 macrophages were higher in the lung affected by cancer when compared with the ‘healthy’ lung and correlated with advanced disease [21,28]. Those findings suggest that the processes of immune regulation and suppression, which are very strong in malignancy, are triggered and stimulated at the tumor site. In this current study, we have investigated the elements of host effector mechanisms which are connected with the persistent antigenic stimulation and develop an individual homeostasis over time and affect the whole airways. Very recently, it was reported that the majority of lung cancer TILs consist of antigen-experienced T cells with a majority of non-tumor specific T cell receptors (TCRs) [39]. A great majority of these T cells are specific for viral epitopes which is consistent with our hypothesis that the BALF-retrieved CD8+ T cells assessed in our study are mainly antigen-experienced effector/memory cells. Together with the abundance of such antigen-experienced T cells in both lungs, it may be an explanation of the lack of significant differences between the two lung compartments. 

The PD-1-PD-L1/PD-L2 pathway has recently become the most common targets for immunotherapy of NSCLC [1,3,40]. We focused on one arm of this system: the PD-1 molecule. PD-1 expression was described on the main T cell populations in the blood and tumor tissue immune cell infiltrates [22,23,25]. Here we precisely show an expression of these molecules on lymphocytes in their different stages of differentiation as a proportion of PD-1+ cells and also as the intensity of expression. Although not significantly, the proportion of CD8+PD-1+ cells was higher in the malignant tumor site when compared to the benign tumor site and the lung free of tumor. Our findings are in agreement with the results of the study of Ahmadzadeh et al. in which the proportion of PD-1+ T cells was higher in the cancer infiltration in comparison to normal tissue or blood [9]. We found some significant differences between the levels of expression of PD-1 in the blood and BALF T cells. Activated and memory T cells whose proportions were higher in the BALF had a lower expression of PD-1 than in the PB. We hypothesize that circulating T cells contain recently antigen-activated lymphocytes which highly upregulate PD-1 [41] in contrast to lung-resident T cells, which mostly contain exhausted T cells which present a lower level of PD-1. Interestingly, we observed a significant correlation between activated PD-1+CD8+ cells and other cell types in each compartment: the clBALF, hlBALF, and PB, which reflects preserved immune homeostasis. 

Simultaneously with PD-1, we investigated also the expression pattern of CLTA-4. Both suppressors are the main targets for immunotherapy, both occur together on T cells and the blockers of both have a synergistic and additive effect [8,42,43]. The co-expression of suppressor molecules was observed in many studies [24,25,44]. In our study, the CTLA-4 molecule was found to be expressed in a similar manner to PD-1, especially on activated CD8+ cells. Similar results were presented by others [15,42]. Moreover, we also observed naïve T cells with CTLA-4 expression and a very high intensity of CTLA-4 staining in the PB, which is a relatively new observation. There are two domains of CTLA-4: intercellular and extramembrane [13]. In this study, we focused on the membrane domain, which is functionally relevant in contrast to cytoplasmatic protein [13]. The studies on CTLA-4 molecule in the context of Tregs is much more expanded than of effector T cells [13,21]. Some implications of CTLA-4 expression as a prognostic factor may be taken into account; however, to date, there is a lack of markers for the anti-CTLA4 therapy. Our results may implicate a direction in the investigation of CTLA-4 in the biology of T effector cells in lung cancer. 

The major weakness of our study is the lack of possibility to present a follow-up of the patient group. Also, we are aware that the sample size is low and requires further investigations. However, we carefully analyzed the clinical data and we found significant differences in the proportion of PD-1 and CTLA-4 molecules between squamous cell carcinoma (SCC) and adenocarcinoma (AD). The higher proportion of positive T cells in SCC may result from the higher mutational burden and the influence of smoking. Unfortunately, in the current study, we did not access the mutational status of tumors. Nevertheless, such a relationship is supported by the clinical data—the effectiveness of ICIs was firstly documented in SCC. In the first study of nivolumab in the neoadjuvant therapy it was shown to be effective in tumors with a high pre-treatment mutational burden [45], however in the recently published results of combination therapy: nivolumab plus ipilimumab better results were achieved in non-SCC [43]. Patients with EGFR gene mutations common in adenocarcinoma have a low benefit from the ICIs therapy [46], what may confirm the differences between SCC and AD. A prospective observational study should be performed to evaluate the prognostic utility of BALF analysis in the immunotherapy of lung cancer.

## 4. Patients and Methods

### 4.1. Patients

The study group consisted of 32 patients consecutively enrolled during diagnostic procedures of lung tumor. All the patients underwent a clinical examination, bronchoscopy with BALF (the Department of Surgery, the National Institute of Tuberculosis and Lung Diseases, Warsaw, Poland). Each patient had provided written informed consent before bronchoscopy with BAL (the Medical University of Warsaw Ethics Committee, KB/250/2012, 13 November 2012). We qualified patients without any type of previous or recent anti-cancer therapy, clinical signs of infection, chronic obstructive pulmonary disease (COPD), autoimmune diseases, immunosuppressive treatment. The clinical characteristics of the lung cancer patients is summarized in Table 1. 

All procedures performed in the studies involving human participants were in accordance with the ethical standards of the institutional and/or national research committee and with the 1964 Helsinki declaration and its later amendments or comparable ethical standards. Informed consent was obtained from all individual participants included in the study.

### 4.2. Bronchoalveolar Lavage 

Bronchoalveolar lavage was performed during a routine diagnostic bronchofiberoscopy. To each lung, 100 ml of a 0.9% NaCl solution was instilled: BALF was taken from the cancerous lung (clBALF) and from the ‘healthy’ lung (hlBALF) of the same patient during the same procedure. Two milliliters of blood from each patient was collected at the same time of the day and drawn into tubes with EDTA. 

BALF processing was realized according to the recommendations [29]. The volume of BAL fluid recovery was 50% or more. The material was filtered through nylon gauze and then the fluid was centrifuged for 10 min (300× *g*). The cell pellets were suspended in 1 mL of phosphate buffered saline (PBS) and 200 µL was gently spread on slide. The Bürker chamber was used to measure the total cell count. The differential cell count was determined on two slides stained with May–Grunwald–Giemsa (MGG) with the use of light microscopy. Additionally, staining was hematoxylin-eosin for malignant cells detection. The cell pellets were used for analysis by flow cytometry.

### 4.3. Flow Cytometry Analysis

Flow cytometry was used for a lymphocyte subtypes analysis in the clBALF, hlBALF, and PB. 100 µL of BALF re-suspended cells or peripheral blood was used for analysis. The proportion of CD4+ or CD8+ subpopulations were determined by a panel of monoclonal antibodies anti: CD4-APC-Cy7, CD8-PerCP, CD3-BV510, CD69-PE, CD127-BV421, and CD45RA-PE-Cy7 (BD, San Jose, CA, USA). The expression of PD-1 and CTLA-4 molecules was evaluated by the use of CD279 (PD-1)-BB515 and CD152 (CTLA-4)-APC (BD, USA) antibodies, respectively. BD FACS™ lyzing solution was used for lysing red blood cells following direct immunofluorescence staining of human peripheral blood cells with monoclonal antibodies prior to the flow cytometric analysis. All cells were analyzed in one tube. The samples were processed by the flow cytometer FACS Canto II (BD, USA). At least 50,000 cells in the lymphocyte gate was collected. The geometric mean fluorescence (GMF) intensity of PD-1 and CTLA-4 on T cells were measured.

The subpopulations of CD8+ and CD4+ cells were defined as follows (all cells were CD3+):

naïve CD8+cells (CD8+CD127+CD45RA+), CD4+cells (CD4+CD127+CD45RA+),

memory CD8+cells (CD8+CD127+CD45RA-), CD4+cells (CD4+CD127+CD45RA-),

activated CD8+ cells (CD8+CD69+CD127-CD45RA-), CD4+ cells (CD4+CD69+CD127-CD45RA-),

activated memory CD8+ cells (CD8+CD69+CD127+CD45RA-), CD4+ cells (CD4+CD69+CD127+CD45RA-).

The proportion of CD8 or CD4 positive cells was presented as a percentage in the lymphocyte gate. The proportion of each subpopulation was presented as a percentage of CD8 or CD4 positive cells. The same concerns the proportion of each subpopulation with PD-1 or CTLA-4 expression. 

### 4.4. Statistical Analysis

The Statistica 12.0 software package (StatSoft Inc., Tulsa, OK, USA) was used for a statistical analysis. For group comparison, the Mann–Whitney U test and Kruskal–Wallis with the post-hoc Wilcoxon’s signed rank test were used. Results were given as the median and interquartile range (P25–P75). A *p* < 0.05 was considered as statistically significant. The correlations between the variables were analyzed with the Spearman’s rank test. The correlations with both *r* ≥ 0.3 and *p <* 0.05 were considered relevant. 

## 5. Conclusions

In conclusion, we found important differences in PD-1 and CTLA-4 expression on CD8+ and CD4+ cells depending on the status of their activation in the lung cancer microenvironment. Moreover, we show a differential expression of checkpoint molecules on peripheral and lung lymphocytes which vary depending on the histopathological type of cancer. We confirm the potential role of BAL procedure in selecting patients for immunotherapy by the analysis of the profile of BALF T cells. However, a further prospective study evaluating the prognostic utility of BALF T cells in the ICIs therapy is required. 

## Figures and Tables

**Table 1:** Characteristics of the study population with lung cancer.

Variable	Patients
Sex F/M (*n*)	12/9
Age (mean ± SD years)	66.8 ± 7.6
Women (mean ± SD years)	66.8 ± 7.6
Men (mean ± SD years)	66.7 ± 7.5
Smoking history	
Smokers/ex-smokers/never-smokers (*n*, %)	14 (76.2%)/5 (23.8%)/2 (9.5%)
Pack/years (mean ± SD)	38.9 ± 16.3
Histology (*n*, %)	
Squamous cell carcinoma	10 (47.6%)
Adenocarcinoma	7 (33.3%)
Large cell	2 (9.5%)
NOS	2 (9.5%)
Cancer grade	G2-6 (26%)
	G3-5 (21%)
	NA-53%
EGFR mutation, ALK rearrageemnt	Non confrimed
Stage of disease (*n*,%)	
IA	6 (28.6%)
IB	7 (33.3%)
IIB	4 (19.0%)
IIIA	4 (19%)
Metastases	0
Symptoms of the respiratory system (interview of the patient) (yes/no (*n*, %))	11 (52.4%)/10 (47.6%)
Cough	13 (61.9%)/8 (38.1%)
Hemoptysis	0 (0.0%)/21 (100.0%)
Dyspnea	16 (76.2%)/5 (23.8%)
One year follow-up	
Surgical resection	16 (69%)
Progression	1 (5%)
Death	0
No data	6 (26%)

Abbreviations: CRP, C-reactive protein; F, female; Hb, hemoglobin; LDH, lactate dehydrogenase; M, male; NOS, not otherwise specified; SD, standard deviation; WBC, white blood cells. No routine PD-L1 detection was performed in these years of patient’s enrollment.

**Table 2:** Lymphocyte subtypes in patients with lung cancer. Comparison of the proportion of cells between three compartments: the tumor environment clBALF, ‘healthy’ lung (hlBALF) and peripheral blood (PB). Data expressed as median (p25–p75). * *p* < 0.05.

Cell Type	A. clBALF *n* = 21	B. hlBALF *n* = 21	C. PB *n* = 21	*p* < 0.05 * Group A-B-C ANOVA, Kruskal-Wallis	*p* < 0.05 * Group in Groups Post-Hoc
Lymphocytes	17.4 (10.2–22.1)	17.1 (12.8–19.7)	30.7 (25.9–36.3)	0.0003	A-C: 0.0009
					B-C: 0.0023
T cells CD3+ (% of all cells)	7.7 (4.5–9.4)	7.9 (6.4–12.6)	16.4 (13.5–26.4)	<0.0001	A-C: <0.0001
					B-C: 0.0028
T cells CD3+ (% of all lymphocytes)	59.0 (52.2–67.8)	58.4 (36.3–67.3)	59.0 (52.2–67.8)	0.1689	-
CD8+ (% of T cells)	30.2 (22.4–37.0)	33.7 (23.4–44.1)	29.8 (25.6–37.7)	0.7782	-
CD4+ (% of T cells)	24.2 (15.0–38.9)	19.7 (14.9–29.1)	57.8 (48.7–64.1)	<0.0001	A-C: <0.0001
					B-C: <0.0001
Ratio CD4: CD8	0.8 (0.5–1.3)	0.6 (0.4–1.3)	2.1 (1.3–2.4)	0.0003	A-C: 0.0037
					B-C: 0.0008
CD8+ subpopulation: (% of CD8+ cells)					
Naïve CD8+ (CD8+CD45RA+CD127+)	4.3 (2.5–7.8)	3.9 (1.4–8.3)	28.3 (25.3–48.4)	<0.0001	A-C: <0.0001
					B-C: <0.0001
Memory CD8+ (CD8+CD127+CD45RA−)	53.9 (34.9–62.1)	50.4 (29.1–57.9)	29.3 (17.3–42.1)	0.0120	A-C: 0.0011
					B-C: 0.0347
Activated CD8+ (CD8+CD69+CD127−CD45RA−)	92.3 (86.3–93.9)	86.8 (83.4–94.4)	26.0 (11.7–38.9)	<0.0001	A-C: <0.0001
					B-C: <0.0001
Activated memory CD8+ (CD8+CD69+CD127+CD45RA)	54.8 (38.0–67.1)	47.3 (29.7–63.5)	3.1 (1.4–4.7)	<0.0001	A-C: <0.0001
					B-C: <0.0001
CD4+ subpopulation: (% of CD4+ cells)					
Naïve CD4+ (CD4+CD45RA+CD127+)	8.8 (5.7–25.0)	7.3 (3.7–12.1)	23.7 (17.0–35.5)	0.0060	-
					B-C: 0.0055
Memory CD4 + (CD4+CD127+CD45RA−)	33.9 (25.0–49.6)	40.7 (30.0–46.0)	16.2 (9.1–21.0)	0.0341	-
					B-C: 0.0505
Activated CD4+ (CD4+CD69+CD127−CD45RA−)	74.0 (66.7–78.6)	73.8 (59.0–82.3)	8.9 (7.2–14.2)	<0.0001	A-C: <0.0001
					B-C: <0.0001
Activated memory CD4+ (CD4+CD69+CD127+CD45RA)	35.0 (19.7–47.1)	37.1 (27.3–51.8)	3.7 (1.8–5.9)	<0.0001	A-C: <0.0001
					B-C: <0.0001

**Table 3:** Proportion of lymphocyte subtypes with the expression of PD-1 in patients with lung cancer and the geometric mean fluorescence (GMF) intensity of PD-1 on CD8, CD4 lymphocyte subpopulations. Comparison of the proportion of cells between three compartments: the tumor environment clBALF, ‘healthy’ lung (hlBALF), and peripheral blood (PB). Data expressed as median (p25–p75). Differences between groups were assessed by the ANOVA Kruskal–Wallis test. * *p* < 0.001 between given compartment and peripheral blood.

Lymphocyte Subset (%) Median (p25–p75)	A. clBALF *n* = 21	B. hlBALF *n* = 21	C. PB *n* = 21	*p* < 0.05 * Group A-B-C ANOVA, Kruskal-Wallis	*p* < 0.05 * Group, in Groups Post-Hoc
CD8+ subpopulation: (% of CD8+ cells)					
all CD8+PD1+ (CD8+PD1+)	68.1 (50.6–79.3)	51.8 (40.9–78.7)	25.9 (20.2–33.8)	<0.0001	A-C: <0.0001
					B-C: 0.0001
naïve CD8+ PD-1+ (CD8+CD45RA+CD127+PD-1+)	1.5 (1.1–3.3)	1.8 (0.5–4.3)	3.7 (2.4–5.9)	0.0608	-
memory CD8+ PD-1+ (CD8+CD127+CD45RA-PD-1+)	33.8 (24.0–54.2)	22.9 (13.5–44.0)	11.0 (5.5–14.5)	<0.0001	A-C: <0.0001
					B-C: 0.0002
activated CD8+ PD-1+ (CD8+CD69+CD127-CD45RA-PD-1+)	64.7 (47.1–75.1)	48.4 (37.5–70.2)	10.2 (6.4–19.4)	<0.0001	A-C: <0.0001
					B-C: <0.0001
activated memory CD8+ PD-1+ (CD8+CD69+CD127+CD45RA-PD1+)	30.9 (23.7–57.0)	26.9 (18.1–53.0)	2.8 (1.1–4.4)	<0.0001	A-C: <0.0001
					B-C: <0.0001
GMF					
all CD8+PD1+ (CD8+PD1+)	2202 (1890–2530)	2007 (1793–2431)	2158 (1769–2580)	0.5683	-
naïve CD8+ PD-1+ (CD8+CD45RA+CD127+PD-1+)	1825.5 (1489.5–2070)	1886 (1376–2431)	1500 (1354–1707)	0.0139	A-C: 0.0406
					B-C: 0.0313
memory CD8+ PD-1+ (CD8+CD127+CD45RA-PD-1+)	2283 (1933–2685)	2248 (1746–2556)	2261 (2003–2646)	0.7570	-
activated CD8+ PD-1+ (CD8+CD69+CD127-CD45RA-PD-1+)	2267 (2013–2677)	2151 (1843–2444)	3416 (3043–3694)	0.0001	A-C: 0.0023
					B-C: 0.0001
activated memory CD8+ PD-1+ (CD8+CD69+CD127+CD45RA-PD1+) Geo Mean	2476 (1974–2771)	2289 (1918–2572)	4282 (4013–4472)	<0.0001	A-C: 0.0001
					B-C: <0.0001
CD4+ subpopulation: (% of CD4+ cells)					
all CD4+PD1+ (CD4+PD1+)	52.3 (44.3–68.6)	54.8 (45.4–68.4)	25.0 (16.3–37.2)	<0.0001	A-C: <0.0001
					B-C: <0.0001
naïve CD4+ PD-1+ (CD4+CD45RA+CD127+PD-1+)	4.9 (1.5–10.1)	2.0 (0.5–4.9)	0.8 (0.3–1.8)	0.0013	A-C: 0.0008
					A-B:0.07
memory CD4+ PD-1+ (CD4+CD127+CD45RA-PD-1+)	61.0 (51.0–82.2)	66.5 (54.9–75.5)	51.7 (41.2–57.4)	<0.0001	A-C: 0.0001
					B-C: <0.0001
activated CD4+ PD-1+ (CD4+CD69+CD127-CD45RA-PD-1+)	46.6 (38.9–64.8)	49.4 (40.5–65.1)	7.4 (3.3–12.0)	<0.0001	A-C: <0.0001
					B-C: <0.0001
activated memory CD4+ PD-1 (CD4+CD69+CD127+CD45RA-PD1+)	23.4 (14.4–37.7)	30.7 (18.2–35.8)	3.6 (1.8–5.6)	<0.0001	A-C: <0.0001
					B-C: <0.0001
GMF					
all CD4+PD1+ (CD4+PD1+)	2471 (2071–3036)	2658 (2271–3274)	2511 (2133–2626)	0.2275	-
naïve CD4+ PD-1+ (CD4+CD45RA+CD127+PD-1+)	1797 (1665–2034)	1867 (1693–2134)	1759 (1575–1957)	0.5243	-
memory CD4+ PD-1+ (CD4+CD127+CD45RA-PD-1+)	2775 (2331–3323)	2737 (2635–3325)	2310 (2076–2495)	0.0019	A-C: 0.0196
					B-C: 0.0026
activated CD4+ PD-1+ (CD4+CD69+CD127-CD45RA-PD-1+)	2699 (2311–3304)	2839 (2423–3424)	4933 (4631–5426)	<0.0001	A-C: <0.0001
					B-C: 0.0001
activated memory CD4+ PD-1 (CD4+CD69+CD127+CD45RA-PD1+)	3271 (2551–3682)	3117 (2799–3780)	5112 (4899–5314)	<0.0001	A-C: <0.0001
					B-C: <0.0001

**Table 4:** Proportion of lymphocyte subtypes with the expression of CTLA-4 and the geometric mean fluorescence (GMF) intensity of CTLA-4 on CD8, CD4 lymphocyte subpopulations in patients with lung cancer. Comparison of the proportion of cells between three compartments: the tumor environment clBALF, ‘healthy’ lung (hlBALF) and peripheral blood (PB). Data expressed as median (p25–p75).

Lymphocyte Subset (%) Median (p25–p75)	A. clBALF *n* = 21	B. hlBALF *n* = 21	C. PB *n* = 21	*p* < 0.05 * Group A-B-C ANOVA, Kruskal-Wallis	*p* < 0.05 * Group, in Groups Post-Hoc
CD8+ subpopulation (% of CD8+ cells)					
all CD8+CTLA-4+ (CD8+CTLA-4+)	57.9 (45.6–76.3)	52.6 (37.9–80.7)	15.6 (2.8–46.3)	0.0001	A-C: 0.0002
					B-C: 0.0005
naïve CD8+ CTLA-4+ (CD8+CD45RA+CD127+CTLA-4+)	2.7 (1.0–6.4)	2.7 (0.7–6.0)	3.1 (0.2–12.2)	0.9720	-
					-
memory CD8+ CTLA-4+ (CD8+CD127+CD45RA-CTLA-4+)	33.7 (17.4–44.2)	26.1 (13.4–43.1)	3.7 (1.8–8.5)	<0.0001	A-C: <0.00001
					B-C: <0.0001
activated CD8+ CTLA-4+ (CD8+CD69+CD127-CD45RA-CTLA-4+)	49.4 (40.9–71.0)	48.4 (33.4–75.5)	2.9 (0.3–13.1)	<0.0001	A-C: <0.0001
					B-C: <0.0001
activated memory CD8+ CTLA-4+ (CD8+CD69+CD127+CD45RA-CTLA-4+)	35.7 (21.0–48.3)	25.7 (15.9–47.5)	0.2 (0.1–1.3)	<0.0001	A-C: <0.0001
					B-C: <0.0001
GMF					
all CD8+CTLA-4+ (CD8+CTLA-4) Geo Mean	859 (832–963)	839 (794–1001)	2371 (2279–3311)	<0.0001	A-C: <0.0001
					B-C: <0.0001
naïve CD8+ CTLA-4+ GMF (CD8+CD45RA+CD127+CTLA-4+) Geo Mean	996.5 (889.5–1225.5)	926 (869–1251)	2393 (2236–2692)	<0.0001	A-C: <0.0001
					B-C: <0.0001
memory CD8+ CTLA-4+ GMF (CD8+CD127+CD45RA-CTLA-4+) Geo Mean	898 (850–934)	881 (828–1014)	2482 (2357–3500)	<0.0001	A-C: <0.0001
					B-C: <0.0001
activated CD8+ CTLA-4+ GMF (CD8+CD69+CD127-CD45RA-CTLA-4+) Geo Mean	873 (848–973)	860 (791–1022)	2477 (2318–3231)	<0.0001	A-C: <0.0001
					B-C: <0.0001
activated memory CD8+ CTLA-4+ GMF (CD8+CD69+CD127+CD45RA-CTLA-4+) Geo Mean	921 (863–983)	903 (822–1042)	2506 (2363–3328)	<0.0001	A-C: <0.0001
					B-C: <0.0001
CD4+ subpopulation (% of CD4+ cells)					
all CD4+CTLA-4+ (CD4+CTLA-4+)	98.5 (92.1–99.2)	99.6 (96.8–99.9)	100.0 (99.9–100.0)	0.0689	-
naïve CD4+ CTLA-4+ (CD4+CD45RA+CD127+CTLA-4+)	8.3 (5.4–16.1)	7.1 (3.6–11.4)	23.7 (17.0–35.4)	0.0009	A-C: 0.0126
					B-C: 0.0013
memory CD4+ CTLA-4+ (CD4+CD127+CD45RA-CTLA-4+)	60.4 (51.0–82.1)	66.1 (54.8–75.0)	51.7 (41.2–57.4)	0.0505	-
activated CD4+ CTLA-4+ (CD4+CD69+CD127-CD45RA-CTLA-4+)	69.5 (65.0–78.1)	73.5 (57.8–80.0)	8.9 (7.2–14.2)	<0.0001	A-C: <0.0001
					B-C: <0.0001
activated memory CD4+ CTLA-4+ (CD4+CD69+CD127+CD45RA-CTLA-4+)	35.0 (16.7–47.1)	37.1 (27.3–51.7)	3.7 (1.8–5.9)	<0.0001	A-C: <0.0001
					B-C: <0.0001
GMF					
all CD4+CTLA-4+ (CD4+CTLA-4+) Geo Mean	2555 (2037–3405)	3134 (2236–3547)	6451 (4783–7990)	<0.0001	A-C: <0.0001
					B-C: <0.0001
naïve CD4+ CTLA-4+ GMF (CD4+CD45RA+CD127+CTLA-4+) Geo Mean	1523 (1269–2210)	1751 (1270–2297)	5702 (4651–7905)	<0.0001	A-C: 0.0037
					B-C: 0.0008
memory CD4+ CTLA-4+ GMF (CD4+CD127+CD45RA-CTLA-4+) Geo Mean	3059 (2400–3970)	2801 (2221–3969)	6730 (5082–8560)	<0.0001	A-C: <0.0001
					B-C: <0.0001
activated CD4+ CTLA-4+ GMF (CD4+CD69+CD127-CD45RA-CTLA-4+) Geo Mean	2580 (2208–3591)	3093 (2230–3604)	6744 (4848–8264)	<0.0001	A-C: <0.0001
					B-C: <0.0001
activated memory CD4+ CTLA-4+ GMF (CD4+CD69+CD127+CD45RA-CTLA-4+) Geo Mean	3058 (2529–4358)	3347 (2569–4187)	6934 (5145–8633)	<0.0001	A-C: <0.0001
					B-C: <0.0001

## Data Availability

The datasets used and/or analyzed during the current study are available from the corresponding author on reasonable request.

## Supplementary Materials

The following are available online at https://www.mdpi.com/2072-6694/11/4/567/s1, Figure S1: Scatter plots and histograms with gating strategy for CD8+ cells subpopulations from the bronchoalveolar lavage fluid from the lung affected by cancer (clBALF), Figure S2: Subpopulations of PD-1-positive T cells in lung cancer, Figure S3: PD-1 expression on T cells in lung cancer versus T cells in benign lesions, Table S1: Correlations between proportion of activated CD8+/PD-1+ and other cells in the BALF and PB, Table S2: Correlation between the median proportion of CD8+ and CD4+ cells subpopulation with PD-1 or CTLA-4 expression and smoking history expressed as pack years smoked, Table S3: Comparison of lymphocyte subtypes proportion with PD-1 and CTLA-4 expression in patients with lung cancer and benign tumor, Table S4: Proportion of lymphocyte subtypes with PD-1 and CTLA-4 expression in patients with benign lesions in the BALF from tumor site (clBALF), opposite lung (hlBALF) and peripheral blood (PB).

## Abbreviations

BALF—Bronchoalveolar lavage fluid; clBALF—Cancer-affected lung BALF; ICIs—Check-point inhibitors; CTLA-4—Cytotoxic T cell antigen 4; DCs—Dendritic cells; hlBALF—Healthy symmetrical lung BALF; MGG—May–Grunwald–Giemsa staining; NK—Natural killer cells; NOS—Not otherwise specified; NSCLC—Non-small cell lung cancer; PB—Peripheral blood; PD-1—Programmed death-1; TCR—T cell receptor; TIL—Tumor infiltrating lymphocytes; TME—Tumor microenvironment.
