# Supplementary material for: Immunophenotype of T Cells Expressing Programmed Death-1 and Cytotoxic T Cell Antigen-4 in Early Lung Cancer: Local vs. Systemic Immune Response
Source: Cancers (Basel). 2019 Apr 21;11(4):567. doi: 10.3390/cancers11040567 (PMC6520959; doi:10.3390/cancers11040567)
Supplement: Supplementary file 1 [file cancers-11-00567-s001.pdf]

cancers

Supplementary Material: Immunophenotype of T Cells Expressing Programmed Death-1 and Cytotoxic T Cell Antigen-4 in Early Lung Cancer: Local vs. Systemic Immune Response

Iwona Kwiecien, Tomasz Skirecki, Małgorzata Polubiec-Kownacka, Agata Raniszewska and Joanna Domagala-Kulawik

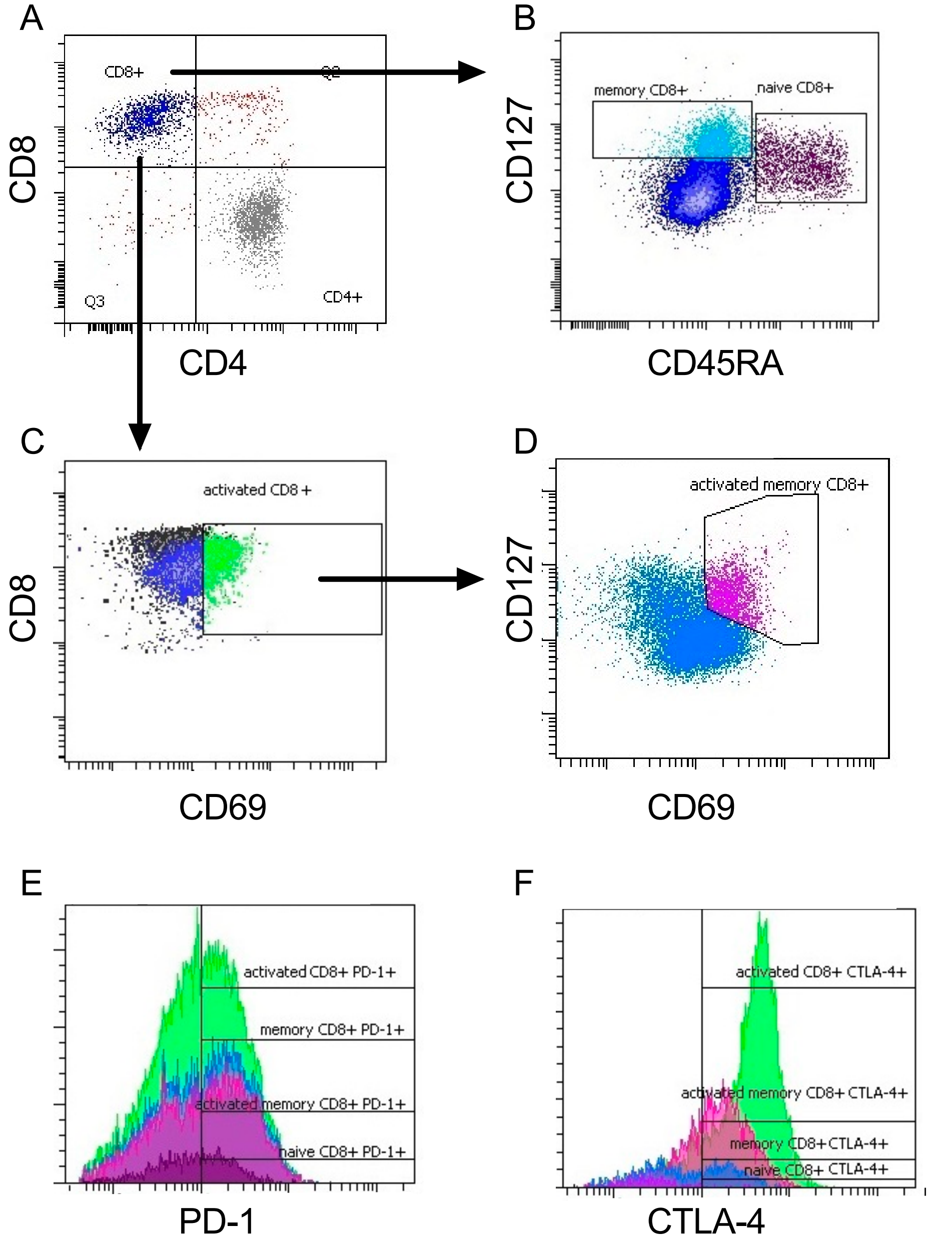

Figure S1. Scatter plots and histograms with gating strategy for CD8+ cells subpopulations from the bronchoalveolar lavage fluid from the lung affected by cancer (clBALF). (**A**) Scatter plot showing CD3+ lymphocytes. (**B**) Gating of CD8+ naive and memory T cells. (**C**) Gating of activated CD8+ T cells. (**D**) Scatter plot from C (CD8+CD69+) for analysis of activated memory CD8 T cells. (**E**) Expression of the PD-1 on the aboventioned CD8+ subpopulations. (**F**) Expression of CTLA-4 on the abovementioned CD8+ subpopulations.

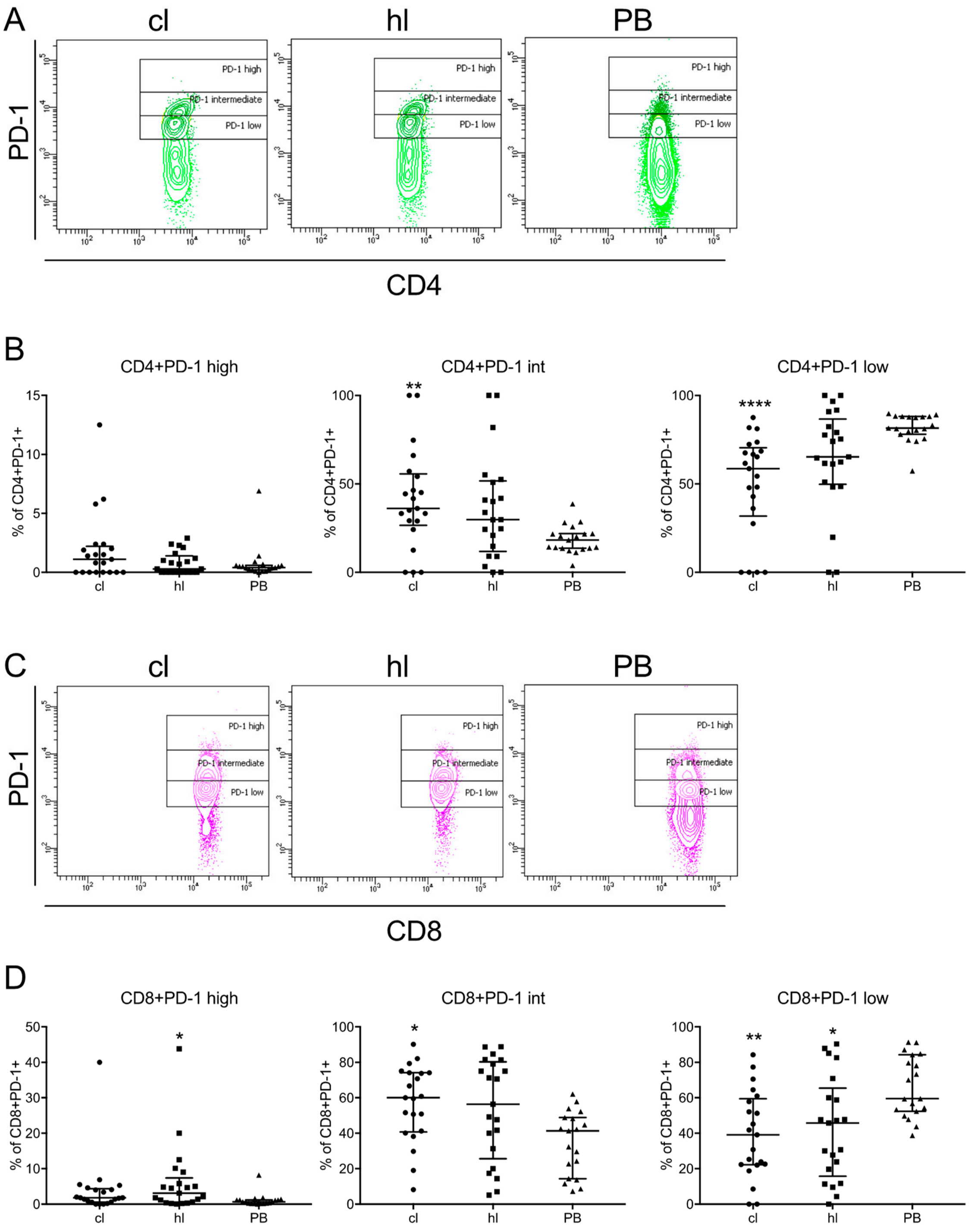

Figure S2. Subpopulations of PD-1-positive T cells in lung cancer. (**A**) Representative staining of PD-1 on CD4+PD-1+ cells of the BALF form cancer affected lung (cl), healthy lung (h) and peripheral blood (PB). (**B**) comparison of the proportions of subpopulations of CD4+PD-1+ T cells. (**C**) representative dot plots of CD8+PD-1+ cells. (**D**) comparison of the proportions of subpopulations of CD8+PD-1+ T cells at different sites. Differences between groups were assessed by the Anova Kruskal-Wallis test. * $p < 0.001$ between given compartment and peripheral blood.

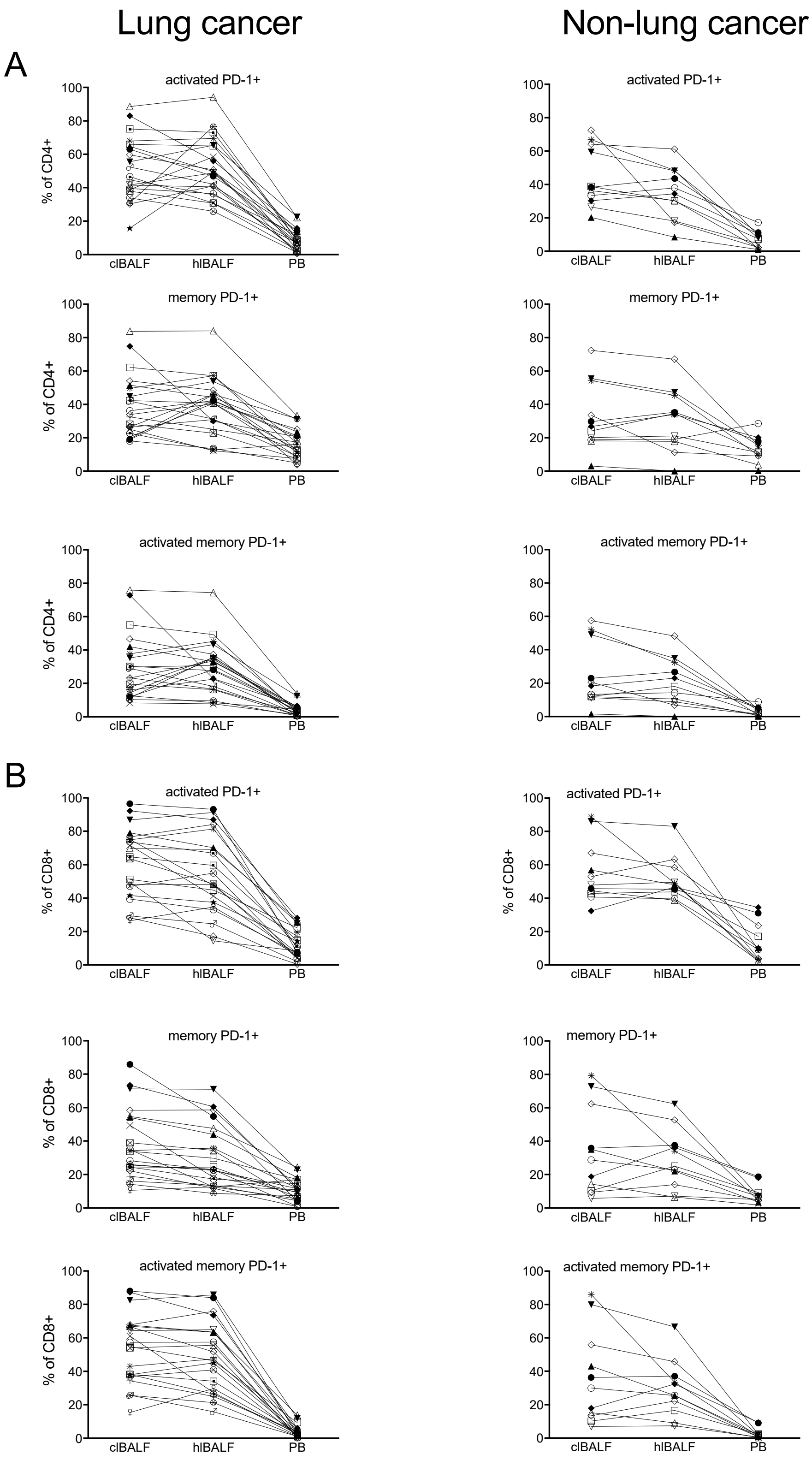

Figure S3. PD-1 expression on T cells in lung cancer versus T cells in benign lesions. (**A**) Proportion of activated, memory and activated memory CD4+ PD-1+ in individual patient- comparison of lung cancer patients with patients with benign lung lesions. (**B**) Proportion of activated, memory and activated memory CD8+ PD-1+ in individual patient- comparison of lung cancer patients with patients with benign lung lesions.

Table S1. Correlations between proportion of activated CD8+/PD-1+ and other cells in the BALF and PB. Significant: $r > 0.3$, $p < 0.05$ in Spearman test.

Events:		Groups: clBALF	hlBALF	PB
Proportion of activated CD8+ PD-1+	CD8+ CTLA-4+	$r = 0.5$	$r = 0.5$	$r = 0.5$
	memory CD8+ PD-1+	$r = 0.7$	$r = 0.8$	$r = 0.6$
	memory CD8+ CTLA-4+	$r = 0.7$	$r = 0.5$	$r = 0.5$
	naïve CD8+ PD-1+	-	$r = 0.5$	$r = 0.6$
	naïve CD8+ CTLA-4+	-	-	$r = 0.4$
	activated CD8+ CTLA-4+	$r = 0.6$	$r = 0.5$	$r = 0.6$
	activated memory CD8+ CTLA-4+	$r = 0.6$	$r = 0.5$	$r = 0.6$
	CD4+ PD-1+	$r = 0.4$	$r = 0.3$	$r = 0.7$
	CD4+ CTLA-4	-	$r = -0.4$	$r = -0.5$
	memory CD4+ PD1+	-	-	$r = 0.6$
	naïve CD4+ PD-1+	$r = 0.4$	$r = 0.5$	$r = 0.7$
	naïve CD4+ CTLA-4+	$r = 0.3$	-	-
	activated CD4+ PD-1+	$r = 0.3$	-	$r = 0.9$
	activated CD4+ CTLA-4	$r = 0.4$	-	$r = 0.7$
	activated memory CD4+ PD-1+	-	-	$r = 0.8$
	activated memory CD4+ CTLA-4	-	-	$r = 0.8$

Table S**2.** Correlation between the median proportion of CD8+ and CD4+ cells subpopulation with PD-1 or CTLA-4 expression and smoking history expressed as pack years smoked. Only significant relations are shown, $p < 0.05$.

Events:		Groups: clBALF	hlBALF	PB
smoking history: Pack years smoked	CD8+ PD-1	$r = -0.3$	-	$r = -0.4$
	activated CD8+ PD-1+	$r = -0.3$	-	$r = -0.3$
	memory CD8+ PD-1+	-	-	$r = -0.6$
	activated memory CD8+ PD-1+	-	-	$r = -0.3$
	naïve CD8+ CTLA-4+	$r = 0.4$	$r = 0.3$	-
	CD4+ cells	$r = 0.3$	$r = 0.5$	-
	CD4+ PD-1+	-	$r = -0.6$	$r = -0.5$
	activated CD4+ PD-1+	-	$r = -0.6$	$r = -0.3$
	memory CD4+ PD-1+	$r = -0.3$	$r = -0.4$	$r = -0.5$
	activated memory CD4+ PD-1+	-	$r = -0.4$	$r = -0.4$
	naïve CD4+ PD-1+	-	$r = 0.4$	-
	CD4+ CTLA-4	-	$r = -0.3$	-
	activated CD4+ CTLA-4	-	$r = -0.5$	$r = -0.3$
	memory CD4+ CTLA-4	-	$r = -0.3$	$r = -0.3$
	activated memory CD4+ CTLA-4+	-	$r = -0.4$	$r = -0.4$
	naïve CD4+ CTLA-4+	-	$r = 0.5$	-
	ratio CD4/CD8	$r = 0.3$	$r = 0.5$	-

Table S3. Comparison of lymphocyte subtypes proportion with PD-1 and CTLA-4 expression in patients with lung cancer and benign tumor. Data expressed as median (p25–p75).

	**Lung Affected by Tumor**		**Lung Without Tumor**	
Lymphocyte subset (%) median (p25–p75)	benign tumor 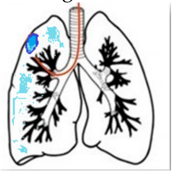	lung cancer (clBALF) 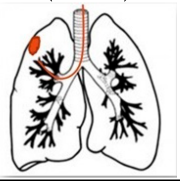	Lung opposite to benign tumor 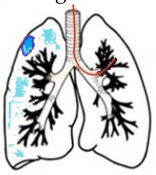	Lung opposite to lung cancer 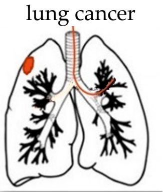
all CD8+PD1+	56.3 (48.6–76.3)	68.1 (50.6–79.3)	55.0 (49.0–67.7)	51.8 (40.9–78.7)
naïve CD8+ PD-1+	1.6 (0.9–3.7)	1.5 (1.1–3.3)	2.2 (0.8–2.4)	1.8 (0.5–4.3)
memory CD8+ PD-1+	28.8 (10.3–62.4)	33.8 (24.0–54.2)	25.0 (14.1–37.7)	22.9 (13.5–44.0)
activated CD8+ PD-1+	47.9 (42.8–67.0)	64.7 (47.1–75.1)	48.0 (43.8–58.4)	48.4 (37.5–70.2)
activated memory CD8+ PD-1+	30.0 (13.4–55.8)	30.9 (23.7–57.0)	25.6 (16.6–37.1)	26.9 (18.1–53.0)
all CD8+ CTLA-4+	60.9 (48.5–76.3)	57.9 (45.6–76.3)	53.2 (43.5–66.7)	52.6 (37.9–80.7)
naïve CD8+ CTLA-4+	3.0 (0.8–7.3)	2.7 (1.0–6.4)	3.1 (1.1–4.4)	2.7 (0.7–6.0)
memory CD8+ CTLA-4+	32.8 (9.7–45.5)	33.7 (17.4–44.2)	28.6 (9.0–43.0)	26.1 (13.4–43.1)
activated CD8+ CTLA-4+	50.9 (32.4–64.6)	49.4 (40.9–71.0)	44.6 (31.5–58.0)	48.4 (33.4–75.5)
activated memory CD8+ CTLA-4+	33.4 (12.4–50.2)	35.7 (21.0–48.3)	29.5 (7.9–37.6)	25.7 (15.9–47.5)
all CD4+PD1+	41.9 (35.4–66.7)	52.3 (44.3–68.6)	41.6 (32.0–53.0)	54.8 (45.4–68.4)
naïve CD4+ PD-1+	2.5 (0.5–9.1)	4.9 (1.5–10.1)	0.8 (0.5–3.0)	2.0 (0.5–4.9)
memory CD4+ PD-1+	26.8 (18.8–54.2)	61.0 (51.0–82.2)	34.0 (17.8 -45.4)	66.5 (54.9–75.5)
activated CD4+ PD-1+	38.4 (30.3–64.1)	46.6 (38.9–64.8)	34.5 (18.1–48.2)	49.4 (40.5–65.1)
activated memory CD4+ PD-1+	18.4 (12.0–49.1)	23.4 (14.4–37.7)	18.0 (8.9–32.4)	30.7 (18.2–35.8)
all CD4+ CTLA-4+	99.1 (94.5–99.8)	98.5 (92.1–99.2)	98.5 (96.4–99.9)	99.6 (96.8–99.9)
naïve CD4+ CTLA-4+	7.2 (4.2–10.2)	8.3 (5.4–16.1)	4.2 (1.6–10.9)	7.1 (3.6–11.4)
memory CD4+ CTLA-4+	51.3 (42.7–80.7)	60.4 (51.0–82.1)	68.0 (37.0–81.0)	66.1 (54.8–75.0)
activated CD4+ CTLA-4+	66.5 (48.9–86.7)	69.5 (65.0–78.1)	63.3 (48.1–69.7)	73.5 (57.8–80.0)
activated memory CD4+ CTLA-4+	25.5 (15.0–59.0)	35.0 (16.7–47.1)	30.5 (16.5–41.4)	37.1 (27.3–51.7)

Table S4. Proportion of lymphocyte subtypes with PD-1 and CTLA-4 expression in patients with benign lesions in the BALF from tumor site (clBALF), opposite lung (hlBALF) and peripheral blood (PB). Data expressed as median (p25–p75).

**Lymphocyte Subset (%) Median (p25–p75)**	**A. bl BALF** *n* **= 11**	**B. hlBALF** *n* **= 11**	**C. PB** *n* **= 11**	***p* < 0,05 * Group A-B-C ANOVA Kruskal-Wallis**	***p* < 0.05 * Group IN GROUPS POST-HOC**
Lymphocytes	29.2 (16.8–37.0)	25.7 (10.2–41.3)	28.3 (25.6–36.7)	0.9288	-
T cells CD3+ (% of all cells)	13.0 (8.4–23.4)	13.2 (4.1–20.3)	19.4 (15.0–23.2)	0.2379	-
T cells CD3+ (% of all lymphocytes)	55.2 (42.8–74.7)	51.8 (37.0–61.6)	59.0 (57.9–66.7)	0.1214	-
CD8+ (% of T cells)	33.5 (16.3–44.7)	26.9 (21.8–41.9)	33.5 (28.3–37.1)	0.5305	-
CD4+ (% of T cells)	23.7 (10.0–40.6)	33.0 (8.3–45.7)	57.5 (40.1–61.8)	0.0167	A-C: 0.0229
Ratio CD4: CD8	0.8 (0.5–1.7)	0.9 (0.5–2.0)	1.7 (1.1–2.2)	0.1657	-
CD8+ subpopulation: (% of CD8+ cells)					
all CD8+PD1+	56.3 (48.6–76.3)	55.0 (49.0–67.7)	23.1 (12.9–42.0)	0.0001	A-C: 0.0007 B-C: 0.0008
all CD8+CTLA-4+	60.9 (48.5–76.3)	53.2 (43.5–66.7)	3.6 (2.2–41.2)	0.0007	A-C: 0.0019 B-C: 0.0042
naïve CD8+	6.4 (1.6–8.7)	3.9 (1.5–8.8)	30.9 (23.8–36.4)	<0.0001	A-C: 0.0003 B-C: 0.0001
naïve CD8+ PD-1+	1.6 (0.9–3.7)	2.2 (0.8–2.4)	3.1 (0.9–11.1)	0.4338	-
naïve CD8+ CTLA-4+	3.0	3.1	0.2	0.5005	-

	(0.8–7.3)	(1.1–4.4)	(0.1–11.2)		-
memory CD8+	45.7 (16.2–75.9)	37.5 (19.3–67.2)	22.7 (16.2–29.4)	0.0503	-
memory CD8+ PD-1+	28.8 (10.3–62.4)	25.0 (14.1–37.7)	6.9 (4.4–9.2)	0.0012	A-C: 0.0039 B-C: 0.0052
memory CD8+ CTLA-4+	32.8 (9.7–45.5)	28.6 (9.0–43.0)	2.0 (1.0–8.7)	0.0004	A-C: 0.0014 B-C: 0.0028
activated CD8+	86.0 (73.9–91.0)	76.8 (70.6–89.3)	20.9 (8.3–38.4)	<0.0001	A-C: 0.0001 B-C: 0.0012
activated CD8+ PD-1+	47.9 (42.8–67.0)	48.0 (43.8–58.4)	9.2 (3.3–23.5)	<0.0001	A-C: 0.0002 B-C: 0.0003
activated CD8+ CTLA-4+	50.9 (32.4–64.6)	44.6 (31.5–58.0)	0.2 (0.0–10.4)	0.0001	A-C: 0.0002 B-C: 0.0014
activated memory CD8+	46.8 (19.0–66.7)	42.5 (20.4–49.5)	1.7 (0.8–2.6)	<0.0001	A-C: 0.0001 B-C: 0.0003
activated memory CD8+ PD-1+	30.0 (13.4–55.8)	25.6 (16.6–37.1)	1.7 (0.7–2.5)	0.0001	A-C: 0.0003 B-C: 0.0005
activated memory CD8+ CTLA-4+	33.4 (12.4–50.2)	29.5 (7.9–37.6)	0.1 (0.0–1.4)	<0.0001	A-C: 0.0001 B-C: 0.0004
CD4+ subpopulation: (% of CD4+ cells)					
all CD4+PD1+	41.9 (35.4–66.7)	41.6 (32.0–53.0)	24.7 (12.5–30.4)	0.0014	A-C: 0.0014 B-C: 0.0297
all CD4+CTLA-4+	99.1 (94.5–99.8)	98.5 (96.4–99.9)	100.0 (99.5–100.0)	0.0611	-
naïve CD4+	8.3 (4.3–19.1)	5.6 (1.6–11.3)	35.4 (24.7–42.6)	0.0013	A-C: 0.0153 B-C: 0.0019
naïve CD4+ PD-1+	2.5 (0.5–9.1)	0.8 (0.5–3.0)	1.8 (0.3–2.3)	0.4300	-
naïve CD4+ CTLA-4+	7.2 (4.2–10.2)	4.2 (1.6–10.9)	30.3 (17.0–39.1)	0.0007	A-C: 0.0105 B-C: 0.0011
memory CD4+	51.4 (42.7–81.1)	69.0 (37.0–81.0)	39.0 (36.4–53.6)	0.1193	-
memory CD4+ PD-1+	26.8 (18.8–54.2)	34.0 (17.8–45.4)	11.6 (9.1–18.1)	0.0093	A-C: 0.0188 B-C: 0.0316
memory CD4+ CTLA-4+	51.3 (42.7–80.7)	68.0 (37.0–81.0)	38.9 (36.4–53.6)	0.1182	-
activated CD4+	67.3 (50–86.7)	64.0 (48.1–69.7)	8.0 (2.4–16.4)	<0.0001	A-C: 0.0001 B-C: 0.0008
activated CD4+ PD-1+	38.4 (30.3–64.1)	34.5 (18.1–48.2)	7.4 (1.9–10.4)	<0.0001	A-C: 0.0001 B-C: 0.0017
activated CD4+ CTLA-4+	66.5 (48.9–86.7)	63.3 (48.1–69.7)	7.6 (2.1–16.3)	0.0002	A-C: 0.0004 B-C: 0.0026
activated memory CD4+	25.5 (15.0–59.1)	30.5 (16.5–41.4)	2.4 (0.8–5.2)	0.0002	A-C: 0.0008 B-C: 0.0017
activated memory CD4+ PD-1	18.4 (12.0–49.1)	18.0 (8.9–32.4)	2.3 (0.8–5.0)	0.0004	A-C: 0.0008 B-C: 0.0045
activated memory CD4+ CTLA-4+	25.5 (15.0–59.0)	30.5 (16.5–41.4)	2.4 (0.8–5.2)	0.0005	A-C: 0.0019 B-C: 0.0023

© 2019 by the authors. Licensee MDPI, Basel, Switzerland. This article is an open access article distributed under the terms and conditions of the Creative Commons Attribution (CC BY) license (http://creativecommons.org/licenses/by/4.0/).
